# Identification of *Trichinella* taxa by ITS-1 amplicon next-generation sequencing with an improved resolution for detecting underrepresented genotypes in mixed natural infections

**DOI:** 10.1186/s13071-023-06035-1

**Published:** 2023-12-21

**Authors:** Vladislav A. Lobanov, Kelly A. Konecsni, W. Brad Scandrett, Emily J. Jenkins

**Affiliations:** 1 Center for Food-borne and Animal Parasitology, Canadian Food Inspection Agency, Saskatoon, SK Canada; 2 Department of Veterinary Microbiology, Western College of Veterinary Medicine, University of Saskatchewan, Saskatoon, SK Canada

**Keywords:** *Trichinella*, Next-generation sequencing, Genotyping method, Wildlife surveillance

## Abstract

**Background:**

Amplicon-based next-generation sequencing (NGS) has rapidly gained popularity as a powerful method for delineating taxa in complex communities, including helminths. Here, we applied this approach to identify species and genotypes of zoonotic nematodes of the *Trichinella* genus. A known limitation of the current multiplex PCR (mPCR) assay recommended by the International Commission on Trichinellosis is that it does not differentiate *Trichinella nativa* from *T. chanchalensis*.

**Methods:**

The new assay entails deep sequencing of an amplified variable fragment of the ribosomal cistron's (rDNA) internal transcribed spacer 1 using the Illumina platform. The assay was evaluated using first-stage larvae (L1) of select laboratory strains of various *Trichinella* taxa mixed in known proportions and then validated using archived L1 from 109 wildlife hosts. The species/genotypes of these L1 isolates from wildlife were previously determined using mPCR.

**Results:**

NGS data analysis for *Trichinella* laboratory strains selected as representative of North American fauna revealed a sequence representation bias. *Trichinella pseudospiralis*, a non-encapsulated species, was the most underrepresented when mixed with *T. spiralis*, *T. murrelli*, *T. nativa* and *Trichinella* T6 in equal quantities. However, five L1 of *T. pseudospiralis* were readily revealed by NGS in a mix with 2000 L1 of *T. nativa* (1:400 ratio). From naturally infected wildlife, all *Trichinella* taxa revealed by mPCR were also identified by NGS in 103 of 107 (96.3%) samples amplified on both assays. NGS identified additional taxa in 11 (10.3%) samples, whereas additional taxa were revealed by mPCR in only four (3.7%) samples. Most isolates comprised single or mixed infections of *T. nativa* and *Trichinella* T6. On NGS, *T. chanchalensis* (T13) was detected in combination with *Trichinella* T6 in a wolverine (*Gulo gulo*) and in combination with *T. nativa* and *Trichinella* T6 in a marten (*Martes americana*) from the Northwest Territories, Canada.

**Conclusions:**

This new NGS assay demonstrates strong potential as a single assay for identifying all recognised *Trichinella* taxa as well as improved sensitivity for detecting under-represented and novel genotypes in mixed infections. In addition, we report a new host record for *T. chanchalensis* in American marten*.*

**Graphical Abstract:**

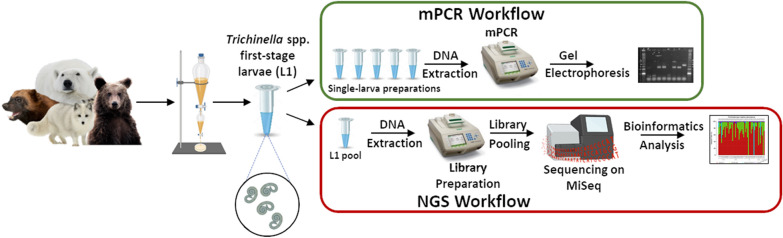

**Supplementary Information:**

The online version contains supplementary material available at 10.1186/s13071-023-06035-1.

## Background

All known taxa of the parasitic nematodes in the genus *Trichinella* are considered capable of causing trichinellosis in humans, a globally significant food-borne zoonotic disease. This zoonosis has been chiefly associated with the consumption of pork, omnivorous or carnivorous wild game and horse meat [1]. The recent discovery of a new member of this genus named *Trichinella chanchalensis* in wolverines from northwestern Canada brought the number of recognised taxa to 13 [2]. Among them are 10 named species and three unnamed genotypes divided into two evolutionary clades characterised by the presence or absence of a collagen capsule surrounding the infective L1. Encapsulated taxa are confined to mammals, whereas non-encapsulated species are found in either mammals and birds or mammals and reptiles [3].

The only species responsible for the domestic cycle involving pigs and synanthropic rats is *T. spiralis* [4]. This species is highly adapted to swine, as substantiated by its high reproductive capacity and prolonged survival of infective L1 in the striated muscle of this host [5]. Epidemiological data suggest that the occurrence of *T. spiralis* in wildlife (sylvatic cycle) is caused mainly by spillover from the domestic cycle [6]. The controlled management conditions under which swine are raised in industrialised countries and regulations for trichinellosis control have substantially reduced the risk of such spillover and acquiring this disease from commercially produced pork. Nowadays, trichinellosis cases in such countries are mainly linked to the consumption of insufficiently cooked or processed game meat (such as wild boar, bear and walrus) harbouring sylvatic *Trichinella* spp. [7]. Except for *T. pseudospiralis*, sylvatic *Trichinella* spp. found in North America have very low infectivity for swine and thus constitute a negligible risk of introduction into commercial pig herds (reviewed in [8]). However, the infectivity of *T. chanchalensis* for swine and other livestock species remains to be determined. Continuous risk-based monitoring of indicator wildlife hosts for *Trichinella* spp. is, therefore, required to support claims of negligible risk status of the commercial swine population [9].

Direct detection of *Trichinella* L1 in striated muscle from known predilection sites by artificial digestion followed by microscopic examination of the resultant sediment is the internationally accepted method for food safety and epidemiological surveillance [10, 11]. However, identifying isolated L1 requires molecular genotyping methods as they are morphologically indistinguishable amongst the different *Trichinella* taxa [12]. A conventional multiplex PCR (mPCR) targeting the internal transcribed spacers 1 and 2 and the large subunit's expansion segment five of rDNA is currently the most widely used method for identifying *Trichinella* spp. [13]. This genotyping method endorsed by the International Commission on Trichinellosis (ICT) enables reliable differentiation of most *Trichinella* taxa. However, ancillary methods are required to distinguish *T. nativa* from *T. chanchalensis* (sympatric in North America) and *T. patagoniensis*, or *T. britovi* from *Trichinella* genotypes T8 and T9 [2, 12]. A real-time PCR with high-resolution melting analysis that differentiated eight *Trichinella* spp. has also been described [14]. However, this assay and mPCR require several individual larva preparations to detect co-infections with two or more *Trichinella* spp. Such co-infections are common in sentinel host species from geographic areas where different *Trichinella* spp. occur in sympatry [15, 16]. However, individual larva preparations are labour-intensive and likely underestimate the prevalences of co-infections with different *Trichinella* spp.

NGS methods may enable a higher sensitivity for detecting underrepresented species in mixed infections. A growing number of published studies have described the species composition of complex parasitic nematode communities using DNA metabarcoding by deep amplicon NGS in various livestock and wildlife host species [17–23]. In the current study, we leveraged this NGS approach for genotyping *Trichinella* spp. and demonstrated its utility in identifying genotypes of these parasitic nematodes isolated from various wildlife hosts in North America and elsewhere, including differentiating the newly discovered *T. chanchalensis* from *T. nativa*, which is currently difficult with commonly used assays.

## Methods

### Parasite material

The laboratory strains of sylvatic *Trichinella* spp. and *T. spiralis* described below have been maintained at the Center for Food-borne and Animal Parasitology (CFAP) by serial passages in CD-1 mice (Charles River) and Sprague-Dawley rats (Charles River), respectively. L1s of these strains were isolated from skinned and eviscerated carcasses of the infected rodents (from 146 to 451 days post-infection) by the pepsin and hydrochloric acid magnetic stirrer artificial digestion method [24] and stored in phosphate-buffered saline (PBS) at − 70 °C until use. Laboratory animal use protocols were approved by the Animal Research Ethics Board of the University of Saskatchewan and adhered to the Canadian Council on Animal Care guidelines for humane animal use. The *T. nativa* strain was initially isolated in 2013 from a naturally infected walrus (*Odobenus rosmarus*) from Nunavik (Quebec, Canada). The *T. murrelli* and *T. pseudospiralis* strains were first isolated from muscle tissue samples of naturally infected cougars (*Puma concolor cougar*) from British Columbia, Canada, received in 2001 and 2004, respectively. The *T. spiralis* strain was isolated in 2013 from meat products from a naturally infected, non-commercially raised domestic pig in southwest Ontario, Canada [25]. *Trichinella* T6 (ISS 40) and *T. britovi* (ISS 107) were initially obtained from the International Trichinella Reference Centre (ITRC) repository (Istituto Superiore di Sanità, Rome, Italy). Additional strains not currently maintained in rodents at CFAP included the *T. murrelli* strain isolated in our laboratory from a naturally infected horse in 2012 [26] as well as *T. murrelli* (ISS 345) and *T. nelsoni* (ISS 29, ISS 37 and ISS 232) from ITRC.

To evaluate the NGS method’s performance using field material, we selected a representative set of archived L1 samples that we previously isolated from various wildlife host species from Canada (Additional file 2: Table S1). The original muscle samples collected from wildlife carcasses, primarily in western and northern Canada, were submitted by federal and provincial agencies or university researchers. Genotyping results generated by mPCR performed as described elsewhere [13] were available for many of these samples. If required and as available, remaining frozen muscle tissues of the source wildlife host were thawed and processed by the artificial digestion method [24] to obtain additional L1. We also assessed L1 isolated from six host species in Russia obtained from the ITRC repository (Additional file 2: Table S1). We focused on larval pools previously genotyped by mPCR as single or mixed infections with *T. nativa* since mPCR cannot differentiate *T. nativa* from potentially co-existing *T. chanchalensis* [2].

To prepare samples with defined larval numbers, L1 is first-stage larvae (plural) of selected pools were counted using a Leica M50 stereo microscope with a magnification of 10 or 16 × . Larvae were manually selected with a pipette from an aliquot in PBS in a petri dish and transferred into microcentrifuge tubes. Tubes with single-larva preparations were examined under the microscope to verify the transfer.

### DNA extraction for the NGS library preparation

Total DNA was extracted from L1 pools (range: 2–100 L1 in a pool) using the PureLink Genomic DNA Mini Kit (Thermo Fisher Scientific, Carlsbad, CA, USA) according to the manufacturer’s protocol for tissue samples. Briefly, PureLink Genomic Digestion Buffer was added to L1 suspended in a small volume of PBS to ~ 180 μl, followed by 20 μl of Proteinase K. The sample was incubated in a thermomixer for 3 h at 56 °C with shaking at 900 revolutions per minute. After the incubation, 20 μl of RNase A was added to the lysate, and the tube was incubated for 2 min at room temperature. After adding 200 μl of PureLink Genomic Lysis/Binding Buffer and 200 μl of 100% ethanol, DNA was purified via spin column and eluted with 50 μl of PureLink Genomic Elution Buffer. A 180-μl aliquot of PBS used for the preparation of larval pools was processed with every batch of samples as a DNA extraction negative control. Purified DNA samples were stored at − 20 °C until use.

### Genotyping of *Trichinella* L1 by mPCR

For a few selected wildlife-derived L1 pools that did not have previously determined mPCR genotyping results, five single larvae and a pool of ≤ 10 L1 were prepared per sample. DNA extraction using the DNA IQ System and Tissue and Hair Extraction Kits (Promega, Madison, WI, USA) and mPCR were performed per ICT recommendations for genotyping *Trichinella* spp. [12]. Each PCR run included a negative DNA extraction control, no template control (i.e., nuclease-free water) and several positive controls extracted from L1 of selected *Trichinella* laboratory strains. A modification of the published procedure entailed using SYBR Gold (Thermo Fisher Scientific) to increase the sensitivity of DNA band visualisation. After electrophoresis, agarose gels were incubated in a working dilution of SYBR Gold in 1 × Tris–borate-EDTA buffer for 1 h (rocked at room temperature) and visualized using the CemiDoc MP Imaging System (Bio-Rad).

### ITS-1 fragment amplification and NGS library preparation

Universal primers were designed with Clone Manager Professional 9.51 (Sci Ed Software) to amplify a variable fragment of ITS-1 of all known *Trichinella* taxa. The size of this fragment varies among reference sequences used in this study from 325 base pairs (bp) for *T. chanchalensis* to 451 bp for *T. pseudospiralis*. Illumina adapter sequences were added to these primers using the approach described elsewhere [17]. The 100 pmol/μl stocks of four forward or reverse primers (Table 1) produced by Integrated DNA Technologies were mixed in equal proportions before use.

**Table 1:** Oligonucleotides used in this study

Primer name	Primer sequence (5′–3′)
ITS-1-F-Adp	TCGTCGGCAGCGTCAGATGTGTATAAGAGACAGCTGCGGAAGGATCATTATCGT
ITS-1-F-Adp1N	TCGTCGGCAGCGTCAGATGTGTATAAGAGACAGNCTGCGGAAGGATCATTATCGT
ITS-1-F-Adp2N	TCGTCGGCAGCGTCAGATGTGTATAAGAGACAGNNCTGCGGAAGGATCATTATCGT
ITS-1-F-Adp3N	TCGTCGGCAGCGTCAGATGTGTATAAGAGACAGNNNCTGCGGAAGGATCATTATCGT
ITS-1-R-Adp	GTCTCGTGGGCTCGGAGATGTGTATAAGAGACAGAACCGTCATGTTGCACAAGTC
ITS-1-R-Adp1N	GTCTCGTGGGCTCGGAGATGTGTATAAGAGACAGNAACCGTCATGTTGCACAAGTC
ITS-1-R-Adp2N	GTCTCGTGGGCTCGGAGATGTGTATAAGAGACAGNNAACCGTCATGTTGCACAAGTC
ITS-1-R-Adp3N	GTCTCGTGGGCTCGGAGATGTGTATAAGAGACAGNNNAACCGTCATGTTGCACAAGTC

Sequences complementary to the target region are underlined

The ITS-1 fragment was amplified in a 25-μl reaction containing 5 μl of 5 × PrimeSTAR GXL buffer (Takara Bio, Kusatsu, Shiga, Japan), 2 μl of 2.5 mM dNTP mix (Takara Bio), 0.75 μl of each 10 pmol/μl forward and reverse primer mix, 0.5 μl of PrimeSTAR GXL DNA Polymerase (Takara Bio), 13.5 μl of nuclease-free water and 2.5 μl DNA template. The cycling protocol consisted of initial denaturation at 98 °C for 2 min followed by 30 cycles of denaturation at 98 °C for 20 s, primer annealing at 60 °C for 15 s and elongation at 68 °C for 30 s. The final extension was performed at 68 °C for 5 min. Each PCR run included a no template control (i.e., nuclease-free water) and DNA extraction negative control. After analysis of PCR results by agarose gel electrophoresis, amplicons were purified using AMPure XP magnetic beads (1 × ; Beckman Coulter, Brea, CA, USA) according to the manufacturer's guidelines and DNA was eluted from beads with 22 μl of 0.1 × TE (1 × TE buffer pH 8 from Thermo Fisher Scientific diluted tenfold with nuclease-free water). After measuring the DNA concentration of each library using the Qubit 3.0 Fluorometer (Thermo Fisher Scientific) and Qubit 1 × dsDNA HS Assay Kit (Life Technologies, Eugene, OR, USA), purified PCR products were diluted with 0.1 × TE to 6.7 ng/μl. Indices and sequencing tags were then added by a limited-cycle PCR using 15 μl (~ 100 ng) of diluted PCR product or undiluted bead-purified reaction mixture for the DNA extraction negative control, 5 μl each of an i7 and i5 oligonucleotide from Nextera XT Index Kit v2 Set A (Illumina, San Diego, CA, USA) and 25 μl of 2 × NEBNext Ultra II Q5 Master Mix (New England Biolabs, Ipswich, MA, USA). The cycling protocol included initial denaturation at 98 °C for 2 min followed by seven cycles of denaturation at 98 °C for 10 s, primer annealing at 55 °C for 30 s and elongation at 65 °C for 1 min and 15 s. The final extension was carried out at 65 °C for 5 min.

Following the purification of amplicons using AMPure XP beads (1 ×), the DNA concentration of each library diluted 1:5 with 0.1 × TE was measured using the Qubit 3.0 Fluorometer as described above. The DNA fragment size distribution analysis was performed using the QIAxcel Advanced System (Qiagen, Hilden, Germany) according to the manufacturer’s guide (NGS Sample Quality Control using the QIAxcel Advanced System, February 2018). The molar concentration of a library was calculated using the following equation.

$$\mathrm{Concentration, nM}=\frac{\mathrm{Concentration,}\frac{\mathrm{ng}}{\mathrm{ \mu L}}}{660\frac{\mathrm{g}}{\mathrm{mol}} \times \mathrm{QIAxcel\, Peak\, Value}} \times 10^6$$

The libraries were diluted to 4 nM with resuspension buffer (10 mM Tris–HCl pH 8.5, 0.1% v/v Tween 20), and 5 μl of each diluted library was pooled. Each preparation of pooled libraries included at least one library prepared from DNA extraction negative control to rule out potential contamination of reagents. After alkaline denaturation, the preparation of pooled libraries was diluted to 8 pM and mixed (at a 3:1 ratio by volume) with denatured PhiX Control v3 (Illumina) diluted to the same concentration. This preparation was incubated at 96 °C for 2 min, followed by 5 min in an ice bath before loading into the reagent cartridge of a MiSeq Reagent Kit v2 500-cycles (Illumina). The MiSeq instrument was set to perform paired-end sequencing and output FASTQ files without post-run analysis. After each sequencing run, the instrument's template line was washed with 0.01% (v/v) sodium hypochlorite to minimise sample carryover from previous runs.

### NGS data analysis workflow

The quality of generated raw reads was assessed using FastQC 0.11.9 (https://www.bioinformatics.babraham.ac.uk/projects/fastqc/). The sequencing data analysis was performed using Geneious 11.1.5 software (Biomatters, Auckland, New Zealand). Paired reads were set for each library from FASTQ files containing demultiplexed forward and reverse reads. The following operations were run as a standardised automated workflow. Illumina adapter and quality trimming and sorting were performed using the BBDuk plugin with minimum quality for ‘Trim Low Quality’ set to 30 and minimum length for ‘Discard Short Reads’ set to 200 bp. After merging paired reads using BBMerge with a merge rate set to ‘High’ and deselecting unmerged reads, quality-filtered merged reads were analysed using the Classify Sequences plugin with sensitivity set to ‘High Sensitivity/Medium’. This plugin performs pairwise alignment of reads to a custom database of reference sequences. The database contained a single manually verified consensus sequence of the ITS-1 fragment for each of the 13 *Trichinella* taxa (Additional file 1). The output summary provides information about the total number and percent of reads mapped to a reference at a specified identity threshold. The minimum overlap identity to classify at the species level was 99% (default value). The minimum percent identity higher than next best result to classify was the default 0.2% unless otherwise stated. For genotyping L1 from wildlife, we applied an arbitrary cut-off represented by a minimum of 0.5% of reads mapped to a reference for assigning taxa in NGS data analysis.

Using sequences found in standard databases, such as ‘Nucleotide collection’ and ‘Whole-genome shotgun contigs’, available on the National Center for Biotechnology Information (NCBI) website, we produced consensus sequences of the ITS-1 fragment for all *Trichinella* taxa except *T. nelsoni* because of a potential misassembly of reads in this region of the *T. nelsoni* whole-genome data set. To address this problem, we amplified ITS-1 from single-larva preparations of *T. nelsoni* strains ISS 29, ISS 37 and ISS 232. Products amplified from five such preparations per strain were purified using AMPure XP beads and subjected to Sanger sequencing at the National Research Council Canada (Saskatoon, Canada). Forward and reverse sequencing reads were assembled into contigs using Clone Manager Professional 9.51, and a consensus sequence was generated after aligning these contigs with the Multiple Alignment using Fast Fourier Transform (MAFFT) plugin in Geneious 11.1.5.

Quality-filtered merged paired reads of selected libraries were further analysed by mapping to the 13 reference sequences in the database using the BBMap plugin and calling variants using FreeBayes with 'Minimum Alternate Fraction' set to 0.15 (Geneious 11.1.5).

### Statistical data analysis

Statistical significance of the difference in the proportion of reads mapped to a reference from the actual proportion of this species in a pool was determined using the Wilcoxon matched-pairs signed rank test, with a *p*-value < 0.05 considered significant using Prism 6.07 software (GraphPad, La Jolla, CA, USA). The degree of agreement between mPCR and NGS on genotyping the L1 pools from wildlife for each identified *Trichinella* genotype was assessed using Cohen's kappa (GraphPad QuickCalcs available at https://www.graphpad.com/quickcalcs/; accessed on 2023–08-10). Kappa values were assigned the following strengths of agreement: < 0.00 no agreement, 0.00–0.20 slight, 0.21–0.40 fair, 0.41–0.60 moderate, 0.61–0.80 substantial and 0.81–1.00 almost perfect agreement [27].

## Results

### Analysis of interspecific variation of the ITS-1 reference sequences

**Fig. 1:**
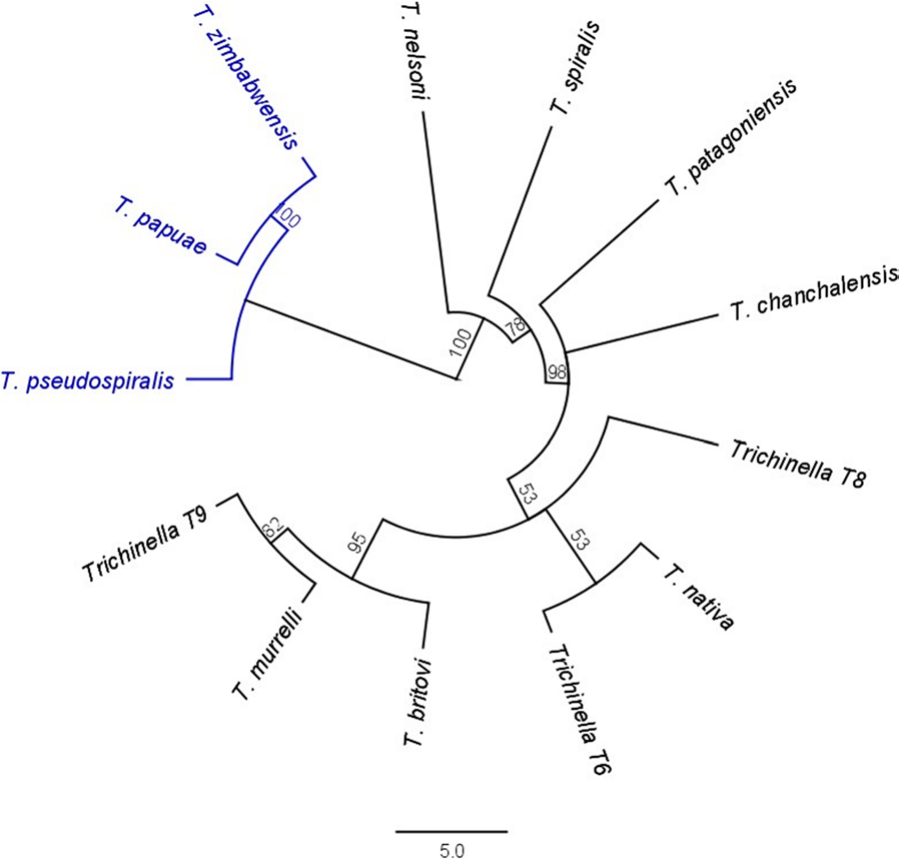
Phylogenetic tree based on consensus sequences of the ITS-1 fragment of 13 *Trichinella* taxa from the custom database used in this study. The sequences were aligned using the MAFFT plugin in Geneious 11.1.5 with default settings. The neighbour-joining method was then applied to produce the tree using the Tamura-Nei model with a bootstrap resampling set at 1000. Bootstrap values are shown at the nodes. Branches of the non-encapsulated clade are depicted in blue

The phylogenetic tree constructed from consensus reference sequences of the ITS-1 marker demonstrates that this fragment possesses sufficient variability for differentiating currently recognised *Trichinella* taxa (Fig. 1). The highest percent identity (99.14%; only three single nucleotide polymorphisms in this fragment) was between *T. murrelli* and *T. britovi* as well as *T. murrelli* and *Trichinella* T9 (Additional file 3: Table S3).

### Performance of the NGS genotyping assay with preparations of mixed laboratory strains of *Trichinella*

**Fig. 2:**
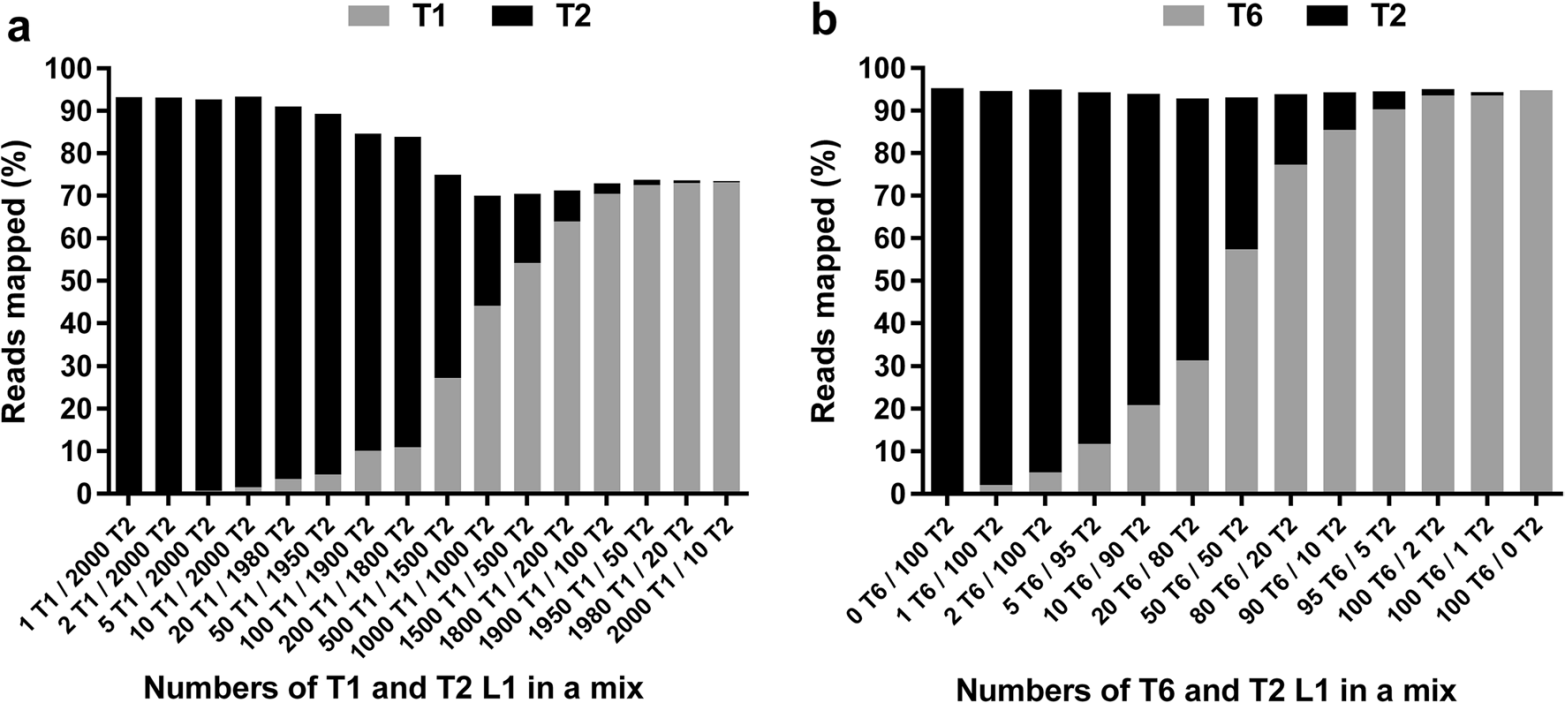
Analysis by the NGS method of larval preparations of two different *Trichinella* taxa mixed in varying proportions. Columns depict the percent of reads mapped to corresponding reference sequences for (**a**) pools of approximately 2000 L1 consisting of different proportions of *T. spiralis* (T1) and *T. nativa* (T2) and (**b**) pools of 100 L1 of *T. nativa* (T2) and *Trichinella* T6 mixed in the numbers indicated

To evaluate the NGS method's performance in identifying co-infecting genotypes, L1 of *T. spiralis* and *T. nativa* laboratory strains mixed at various ratios for a total of approximately 2000 pooled L1 were used for the library preparation. After sequencing, the mean number of paired reads per library was 991,506 (range: 828,602–1,159,000). In the sequencing data analysis, the number of reads mapped to a reference sequence correlated directly with the number of L1 of the respective species in the mix (Fig. 2a). For the libraries prepared from only one, two or five *T. spiralis* L1 in a pool, 0.065%, 0.08% and 0.68% of quality-filtered merged reads, respectively, were mapped to the *T. spiralis* reference sequence. A noticeable sequence representation bias towards *T. spiralis* was illustrated by a higher proportion of reads mapped to the corresponding reference when mixed with an equal number (1000 L1) of *T. nativa* in the pool.

In the next experiment, libraries produced from a series of pools of 100 L1 consisting of different proportions of *T. nativa* and *Trichinella* T6 were sequenced. A lower number of L1 in a pool was used because comparatively low numbers of L1 are typically isolated from wildlife samples. The mean number of paired reads generated per library in this sequencing run was 908,614 (range: 820,348–999,492). There was again a positive correlation between the number of reads mapped to a reference and the number of L1 of the same species in a pool (Fig. 2b). When only a single L1 of *Trichinella* T6 or *T. nativa* was included in a pool, 2.08% and 0.87% of reads were mapped to the respective reference sequences. The sequence representation in this NGS run was biased towards *Trichinella* T6.

We also sequenced this set of libraries using the same version of the MiSeq Reagent kit containing a 'nano' flow cell. The mean number of paired reads per library was 11-fold lower (82,410; Range: 78,064–90,028) than that generated using a ‘standard’ flow cell. However, the proportions of reads mapped to *T. nativa* and *Trichinella* T6 references were similar for each library between the runs generated by either flow cell type, with a difference not exceeding ± 0.8% (Additional file 4: Table S4). In these data sets, the background level represented by the proportion of reads mapped to a non-target reference did not exceed 0.03%.

### Assessment of the sequence representation bias in the NGS assay

**Fig. 3:**
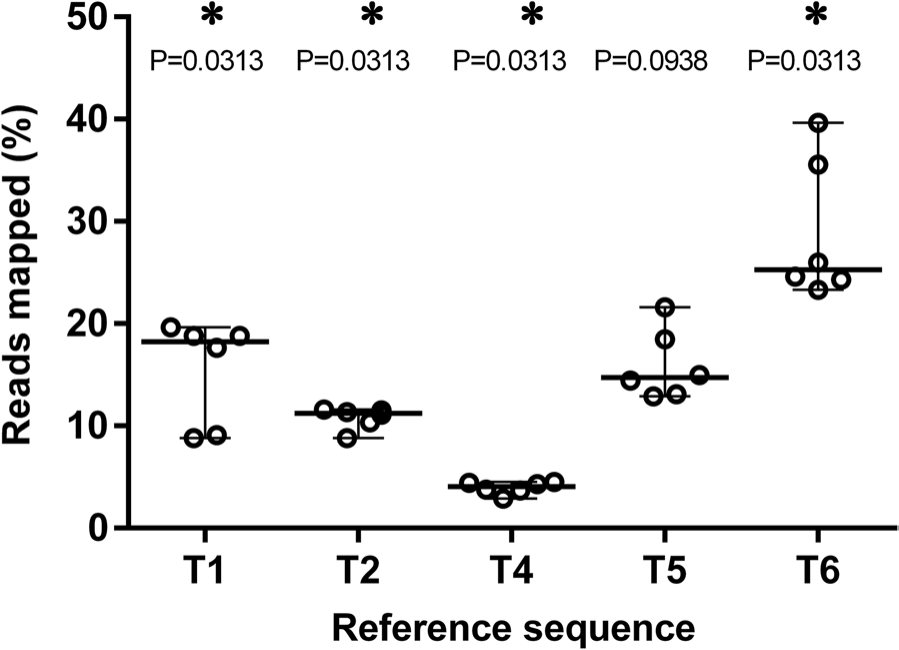
Assessment of the sequence representation bias in the NGS method. Two and four preparations of a pool containing 100 L1 of each of five laboratory strains, *T. spiralis* (T1), *T. nativa* (T2), *T. pseudospiralis* (T4), *T. murrelli* (T5) and *Trichinella* T6, were analysed by the NGS method in two separate runs. Individual values representing the percent of reads mapped to a reference sequence are depicted as a scatter plot with median represented by a solid line and range depicted by bars. The shown *p*-values are exact values. Asterisks indicate statistical significance of the difference in the determined proportion of reads mapped to a reference from the actual proportion (i.e., 20%) of this genotype in a pool

To further assess the sequence representation bias in the NGS genotyping assay, libraries were generated from six preparations containing 100 L1 each of *T. spiralis*, *T. nativa*, *T. pseudospiralis*, *T. murrelli* (from cougar) and *Trichinella* T6. Figure 3 demonstrates a significant deviation of the mean percent of reads mapped to a reference sequence from the actual proportion of the corresponding species in a pool for these *Trichinella* taxa except *T. murrelli*. *Trichinella* T6 was consistently the most overrepresented genotype, whereas *T. pseudospiralis* was the most underrepresented. In preparations of one, two or five L1 of *T. pseudospiralis* mixed with approximately 2000 L1 of *T. nativa,* 0.035%, 0.175% and 0.5% of reads were mapped to the *T. pseudospiralis* reference sequence.

### Identification of available *Trichinella* spp. with the highest homology of the ITS-1 fragment by the NGS assay

Since homology between ITS-1 consensus sequences of *T. britovi* and *T. murrelli* in the custom database was comparatively high, which could reduce the resolution provided by this marker for differentiating these taxa, triplicate 500 L1 sub-samples of larval pools of available laboratory strains of these species (except for the horse-derived *T. murrelli* strain where only 100 L1 per sub-sample were used) were assayed by NGS. For the three *T. murrelli* strains, the mean proportion of reads mapped to the *T. murrelli* reference with standard deviation was 96.9 ± 2.5%. The proportion of reads assigned to non-target taxa was within background levels (i.e., < 0.03%). In contrast, although 96.5 ± 0.17% of reads for the single available *T. britovi* strain were mapped to the corresponding reference sequence, a significant proportion of these reads (3.35 ± 0.16%) also mapped to the *T. murrelli* reference. However, forty single-larva preparations of the same *T. britovi* L1 pool genotyped by mPCR produced a band pattern consistent only with *T. britovi* (not shown), indicating contamination of this laboratory strain with *T. murrelli* was unlikely. To eliminate any cross-talk in classifying reads, sequencing data for *T. britovi* were re-analysed with the default value of 'minimum percent identity higher than next best result to classify' (overlap identity range for considering reference sequences to be equally suitable matches to the query) increased to 0.4%. As a result, 94.41 ± 0.19% of reads were mapped to the *T. britovi* reference, while the number of reads assigned to *T. murrelli* was reduced to the background level. The number of reads unclassified because of conflicting taxonomy increased; however, the values remained comparatively low at 5.56 ± 0.19%.

### Comparing the performance of NGS assay to mPCR using archived L1 samples from 17 different species of wild carnivores

Genotyping results generated by both assays for L1 samples from 107 individual animals are summarised in Table 2. Two L1 samples isolated from a cougar and a wolverine did not amplify in mPCR, but NGS revealed co-infections with two taxa in both cases (Additional file 2: Table S1). All *Trichinella* genotypes identified by mPCR were also identified by the NGS method in 103 (96.3%) samples. Complete agreement between the two genotyping assays was observed in 92 (86%) of the 107 samples. Partial agreement was observed in 11 (10.3%) samples for which additional *Trichinella* taxa were identified by NGS and four (3.7%) samples for which additional taxa were identified by mPCR. Co-infections with multiple (two or three) taxa were revealed in 16 (14.9%) and 24 (22.4%) of the 107 animals by mPCR and NGS, respectively. The kappa coefficient values indicated almost perfect agreement between mPCR and NGS for five identified *Trichinella* taxa, substantial agreement for *T. spiralis* and no agreement for *T. chanchalensis* (Additional file 5: Table S5). The mean proportion of unclassified reads with standard deviation for this data set was 4.17 ± 2.1%. The numbers of mapped reads for DNA extraction negative control libraries included in every NGS run were consistently within background levels.

**Table 2:** Comparison of *Trichinella* genotyping results generated using mPCR and NGS for L1 samples from wildlife and other host species

Host species	Number of animals	Complete agreement	Extra taxa identified by	
			NGS	mPCR
Badger (*Taxidea taxus*)	3	3		
Black Bear (*Ursus americanus*)	2	2		
Brown Bear (*Ursus arctos*)*	2	2		
Cat (*Felis catus*; stray)*	1	1		
Cougar (*Puma concolor cougar*)	25	23	2	
Coyote (*Canis latrans*)	10	10		
Dog (*Canis lupus familiaris*; stray)*	1			1
Fisher (*Pekania pennanti*)	8	6	2	
Grizzly Bear (*Ursus arctos horribilis*)	1	1		
Himalayan Black Bear (*Ursus thibetanus laniger*)*	1	1		
Lynx (*Lynx canadensis*)	1		1	
Marten (*Martes americana*)	1		1	
Raccoon (*Procyon lotor*)	1	1		
Raccoon Dog (*Nyctereutes procyonoides*)*	2	2		
Red Fox (*Vulpes vulpes*)	19	18	1	
Wolf (*Canis lupus*)^§^	20	17		3
Wolverine (*Gulo gulo*)	9	5	4	
Total	107	92 (86%)	11 (10.3%)	4 (3.7%)

^*^L1 isolate(s) from Russia

^§^Includes one L1 isolate from Russia

We sought to clarify discrepancies between results obtained with mPCR and NGS if additional isolated L1 or muscle tissues were still available. For example, for the L1 sample derived from a wolf (*Canis lupus*) in the European part of Russia (ISS 7613) where co-infection with *T. nativa*, *T. britovi* and *T. spiralis* was identified by mPCR performed at ITRC, we found only *T. nativa* and *T. britovi* by both NGS and mPCR in our laboratory. Similarly, where *T. spiralis* and *T. nativa* had been identified from ISS 7607 (a stray dog from Chukotka Autonomous District, Russia) by mPCR performed at ITRC, we identified only *T. nativa* by NGS and mPCR in our laboratory. We also observed a discrepancy between quantitative assessments of *T. nativa* and *T. britovi* proportions in the ISS 7613 L1 pool for the two methods. By mPCR performed in our laboratory, 16 of 22 (72.7%) single-larva preparations were identified as *T. britovi* and the remaining six (27.3%) as *T. nativa*. In contrast, in NGS data analyses for libraries prepared from 100 L1 of this pool or pooled equal DNA aliquots from the 22 single-larva preparations, markedly more reads were assigned to *T. nativa* (82.11% and 65.17%) than *T. britovi* (14.5% and 34.47%), implying preferential amplification of the ITS-1 fragment of *T. nativa*.

### Detecting *T. chanchalensis* in two wild mustelids in Canada by NGS

Although most isolates from Canadian wildlife comprised single or mixed infections of *T. nativa* and *Trichinella* T6 (Table 3), *T. chanchalensis* (84.1% of quality-filtered merged reads) was identified by the NGS method in co-infection with *Trichinella* T6 (2.5%) in a wolverine from the Northwest Territories (NT, Canada) (Additional file 2: Table S1). DNA extracted from L1 of this pool did not amplify in mPCR. Furthermore, *T. chanchalensis* (5.2% of reads) was identified in co-infection with *T. nativa* (15.4%) and *Trichinella* T6 (70.9%) in an American marten from NT. In contrast, only *T. nativa* was identified in this sample by mPCR. We also mapped quality-sorted merged reads of these two libraries to reference sequences in the database using the BBMap plugin, which showed 100% pairwise identity of generated consensus sequences with the *T. chanchalensis* reference. However, variant calling using FreeBayes has elucidated markedly different profiles of lower-frequency ITS-1 variants in *T. chanchalensis* L1 isolated from wolverine and American marten (Table 4).

**Table 3:** *Trichinella* taxa identified by NGS in the 107 L1 pools from wildlife and other host species

	Badger	Black Bear	Brown Bear	Cat	Cougar	Coyote	Dog	Fisher	Grizzly Bear	Himalayan Black Bear	Lynx	Marten	Raccoon	Raccoon Dog	Red Fox	Wolf	Wolverine
Total no.	3	2	2	1	25	10	1	8	1	1	1	1	1	2	19	20	9
T2		1	1	1	12	10	1	3		1			1	1	18	11	5
T5					1												
T6	3	1			9			3									
T1/T2														1			
T1/T2/T4			1														
T2/T3*																1	
T2/T6					3			2	1		1				1	8	4
T2/T6/T13												1					

^*^T. britovi

**Table 4:** Differences in sequence variations of NGS reads mapped to the *Trichinella chanchalensis* reference for the L1 pools from wolverine and American marten

Host species	Variation type	Nucleotide position	Change	AO	Depth*
Marten	Indel	201—202	CA—> Deletion	184	1226
	SNP	160	A—> G	187	1226
	SNP	46	G—> C	187	1226
Wolverine	SNP	289	T—> A	21,741	120,382

^*^Vastly different depths of coverage were achieved due to not only different numbers of quality-filtered merged reads mapped to the *T. chanchalensis* reference between the two host species but also different numbers of total reads generated with the use of MiSeq Reagent kits with 'standard' and 'nano' flow cell

AO, number of alternate observations; SNP, single nucleotide polymorphism

## Discussion

In this study, we developed and evaluated the performance of a new genotyping assay for *Trichinella* using amplicon-based NGS. Significant advantages of this method over other published genotyping assays are (i) the potential for differentiating all currently recognised *Trichinella* taxa without the need for ancillary assays, (ii) enhanced resolution for identifying underrepresented species in a mixed infection and (iii) a higher throughput by removing the need for single-larva preparations and increased multiplexing capability. We demonstrate that this genotyping assay enables accurate qualitative assessment of *Trichinella* taxa composition in a sample. With the cut-off selected for assigning taxa, the NGS method could reliably identify an underrepresented genotype at up to a 1:400 ratio in a larval pool. The new method also demonstrated a quantitative potential for establishing relative abundancies of different *Trichinella* taxa in preparations of mixed laboratory strains. However, quantitative estimates generated by this NGS method for L1 from wildlife hosts should be interpreted in light of preferential amplification of genotypes with the least compromised L1.

Using a representative set of L1 samples isolated from various wildlife host species, NGS performed at least as well as mPCR, which has been the customary method for *Trichinella* genotyping for over two decades [13]. All *Trichinella* taxa revealed by mPCR were also identified by the NGS method in 96.3% (103/107) of these samples, demonstrating high concordance between the two assays. In the remaining four L1 samples, the original mPCR assay identified an additional genotype. This discrepancy could be attributed to the prolonged storage of the frozen samples before testing by the NGS method. NGS outperformed mPCR by detecting additional taxa in 11 samples, including identifying *T. chanchalensis* along with *T. nativa* and *Trichinella* T6 in a larval pool isolated from an American marten from NT, Canada. Unfortunately, no remaining L1 or muscle tissue were available from this animal to perform confirmatory genotyping on additional single-larva preparations using ancillary restriction fragment length polymorphism analysis of an amplified cytochrome b gene fragment, capable of differentiating these three taxa [2]. This finding represents the first record of *T. chanchalensis* infection in a host species other than wolverine. Revealing three *Trichinella* genotypes in the L1 pool from a marten in our study is congruent with a record of co-infection with *T. britovi*, *T. nativa* and *T. spiralis* detected in a pine marten (*Martes martes*) from Lithuania [28]. In a previous wildlife survey conducted in our laboratory, the prevalence of *Trichinella* spp. in 101 American martens from Nunavut and British Columbia was 3% [11]. Regardless of this reported low prevalence, our data support including marten and other mustelids in future surveys on geographic and host ranges of *T. chanchalensis*.

The success rate of PCR genotyping on preparations containing damaged or partially disintegrated larvae can be significantly reduced [11, 15, 29]. Therefore, L1 of co-infecting *Trichinella* taxa having similar integrity is a crucial prerequisite to accurately quantitating their relative abundances by amplicon-based NGS. For example, relative abundances of individual constituents of intestinal parasitic nematode communities in cattle and bison were accurately assessed by amplicon-based NGS using third-stage larvae hatched from excreted eggs via coproculture [17, 18]. However, the larval integrity of each co-infecting *Trichinella* spp. can vary considerably, especially in sequential natural infections that are well separated in time or for taxa that are variably adapted to the host species. *Trichinella* L1 survive in hosts for varying periods with lifespan depending on many factors related to the parasite and the host [30]. Data on the length of larval survival of sylvatic *Trichinella* spp. in live hosts are limited. However, the available evidence supports a range of adaptability of a given *Trichinella* genotype to different host species and different survival rates of distinct genotypes of these parasitic nematodes in a specific host [31, 32]. For example, larvae of *T. britovi* and *T. pseudospiralis* established in experimentally infected pigs remained infective for a limited time and displayed different survival rates [5].

Handling of samples collected for *Trichinella* testing can introduce additional quantification bias. Samples of muscle tissues from wildlife are often frozen before *Trichinella* L1 isolation by artificial digestion. Isolated L1 are also often stored frozen for various periods before further analysis. Freeze damage to L1 can occur, rendering them poorly amplifiable or non-amplifiable by PCR [29]. Larvae of freeze-resistant *Trichinella* spp. are more likely to preserve their integrity after isolation from previously frozen muscle tissues by artificial digestion. This could explain, at least in part, the observed discrepancy between quantitative assessments of proportions of highly freeze-resistant *T. nativa* and less freeze-resistant *T. britovi* in the ISS 7613 L1 pool obtained using mPCR and NGS in this study as well as our lack of detection of *T. spiralis* in samples previously genotyped by mPCR at ITRC (ISS 7607 and ISS 7613). Despite these limitations to accurate results posed by compromised larvae, it is recommended that sample selection should also include such larvae to best ensure NGS identifies all taxa present.

The NGS method also demonstrated sequence representation biases in experiments with well-preserved *Trichinella* laboratory strains mixed at known proportions. Different steps of the NGS genotyping workflow can contribute to this phenomenon, where marker amplification, sequencing and NGS data analysis are the most susceptible stages. One of the few studies that assessed and quantified the effects of such biases demonstrated variation of amplified marker fragment length as a primary contributor to the distortion of relative abundance estimates [33]. In that study, shorter fragments of an artificial marker region were overrepresented compared to longer ones, whereas no significant effect of marker sequence composition on the relative proportion estimates was observed. In addition, this bias was more pronounced on Illumina MiSeq than on two PacBio models. Such preferential amplification of shorter sequences was likely a cause of the *T. pseudospiralis* underrepresentation observed in this study, as the ITS-1 marker fragment of this species is the longest among the taxa used. Alignment of the ITS-1 fragment sequences of 13 *Trichinella* taxa from our custom database (not shown) demonstrated complete homology in binding sites for universal primers used in this study, except a single nucleotide substitution in the reverse primer binding site of the *T. pseudospiralis* ITS-1 fragment located closer to the 5' end of the oligonucleotide sequence. This substitution could also contribute to the observed underrepresentation of *T. pseudospiralis*. Also, loci of rDNA are considered to be amongst the most unstable genomic regions, given their repetitive nature and high transcriptional activity [34]. Although the variation of rDNA copy numbers among *Trichinella* spp. has not been sufficiently studied, it was shown that such copy numbers estimated using whole genome shotgun data varied substantially within and across several other nematode taxa [35]. This phenomenon would further contribute to sequence representation biases in DNA metabarcoding, limiting its quantitative reliability.

The accuracy of taxa delineation by DNA metabarcoding largely depends on the taxonomic resolution provided by selected markers [36]. We chose a variable fragment of ITS-1 because it displayed sufficient interspecific sequence variation for reliable differentiation of even the sibling taxa *T. nativa* and *Trichinella* T6, North America's most commonly observed genotypes in wildlife [11]. However, ITS-1 reference sequences of *T. britovi*, *T. murrelli* and *Trichinella* T9 were 99.1% identical, potentially reducing the resolution provided by this marker for differentiating these taxa. Furthermore, there is evidence of *T. britovi* ITS-1 alleles with even higher homology to *T. murrelli*. The search of the NCBI database using the *T. murrelli* reference sequence as a query generated several hits represented by *T. britovi* isolates from different wildlife hosts in Israel (e.g., KU374884, KU374875) [37] with 99.4% identity of this fragment. Although *T. britovi*, *T. murrelli* and *Trichinella* T9 are geographically well separated [1, 38], reliable differentiation of these taxa would still be desirable. In the sequence analysis of the *T. britovi* laboratory strain performed in this study, a low but significant proportion of generated reads was initially assigned to *T. murrelli*, implying this laboratory strain possesses lower-frequency ITS-1 alleles with higher homology to *T. murrelli* than to the *T. britovi* reference. Although this could be reduced to negligible levels by increasing the overlap identity range value at the sequence classification step of the data analysis workflow, future incorporation of an additional marker region with higher sequence variation between these *Trichinella* spp. might better facilitate their reliable differentiation by the NGS approach.

The efficacy of a marker in delineating species boundaries also depends on the existence of a clear-cut difference between inter- and intraspecific variability [39]. The data generated in this study demonstrated high intraspecific conservation of the ITS-1 marker among *T. nativa* and *Trichinella* T6 isolates from Canadian wildlife. This was substantiated by the following observation. The Classify Sequences plugin in the data analysis workflow assigns quality-filtered reads with minimum overlap identity for references in the database lower than the set threshold into the unclassified reads category. In this study, the proportions of such unclassified reads for all L1 samples, including those isolated from wildlife, were comparatively low, with over 90% of merged reads consistently mapping to one or more reference sequences at the 99% identity threshold, even with only a single reference ITS-1 sequence of each taxon in the database.

For L1 pools with known composition, such as preparations of mixed laboratory strains of *Trichinella*, the ratio of reads mapped to an ITS-1 reference of a non-target genotype was consistently very low (≤ 0.03%). Potential sources of such background in DNA metabarcoding using the Illumina platform include assigning incorrect reads to samples during demultiplexing because of cross-talk caused by misread bases within index sequences and sample carryover from the previous sequencing runs performed on the same instrument [40].

The ability to sequence large numbers of pooled libraries simultaneously or sample multiplexing can offset otherwise comparatively high costs of NGS. Although NGS costs continue to decline, processing low library numbers might still be considered impractical. However, we demonstrated in this study that an adequate sequencing depth could be achieved using the MiSeq Reagent Kit v2 with a 'nano' flow cell, representing a cost-efficient choice when small to moderate sample sizes must be processed. The high coverage depth providing sufficient resolution for determining marker allele composition and frequency is an added value of amplicon NGS, which was previously utilized in genotyping multi-clonal malaria infections [41]. We demonstrated differences in lower abundance ITS-1 variants (within 15–20% of total quality-filtered merged reads) between *T. chanchalensis* isolates from wolverine and American marten. However, this finding should be interpreted cautiously, as distinguishing true haplotypes from sequencing errors may require a more robust approach, including sequencing samples in duplicate [41]. In future studies, we will continue evaluating this feature of the NGS method with more isolates of *T. chanchalensis* and other *Trichinella* taxa.

## Conclusions

We developed and evaluated a method based on deep sequencing of a variable ITS-1 fragment of *Trichinella* spp. on the Illumina platform. The method enabled accurate determination of *Trichinella* taxa composition in larval pools isolated from various host species. We demonstrated excellent overall agreement between the NGS assay and mPCR on genotyping a representative number of L1 isolated from various wildlife hosts. However, the NGS method demonstrated enhanced resolution for detecting underrepresented genotypes in larval pools, including the novel species *T. chanchalensis* not identifiable by mPCR. The performance of the NGS method was demonstrated in this study using strains and isolates of eight of the 13 known *Trichinella* taxa, focusing on those known or most likely to be present in North America, the location of our reference laboratory. However, analysis of sequence data available in GenBank for the other remaining taxa, *T. zimbabwensis*, *T. papuae*, *T. patagoniensis* and *Trichinella* T8 and T9, suggests that the ITS-1 primers will also amplify the marker fragment of these taxa enabling their differentiation by the NGS method.

### Supplementary Information

**Additional file 1. **Reference sequences of the ITS-1 marker fragment of *Trichinella *spp.

**Additional file 2: Table S1**. Comparison of genotyping results generated by mPCR and NGS for selected larval pools isolated from wildlife.

**Additional file 3: Table S3**. ITS-1 fragment identity matrix.

**Additional file 4: Table S4**. Comparison of NGS genotyping results generated using two different types of flow cell.

**Additional file 5: Table S5**. *Trichinella* genotype-specific agreement between mPCR and NGS for the 107 L1 samples from wildlife.

## Data Availability

The raw NGS data generated in this study were deposited at NCBI under BioProject PRJNA911791.

## Abbreviations

Bp: Base pairs
ITS-1: Internal transcribed spacer 1
L1: First-stage larvae
NGS: Next-generation sequencing
PCR: Polymerase chain reaction
mPCR: Multiplex PCR
spp: Species (plural)

## References

[1] Gottstein B, Pozio E, Nockler K (2009). Epidemiology, diagnosis, treatment, and control of trichinellosis. Clin Microbiol Rev.

[2] Sharma R, Thompson P, Hoberg EP, Brad Scandrett W, Konecsni K, Jane Harms N (2020). Hiding in plain sight: discovery and phylogeography of a cryptic species of *Trichinella* (Nematoda: Trichinellidae) in wolverine (*Gulo gulo*). Int J Parasitol.

[3] Zarlenga D, Thompson P, Pozio E (2020). *Trichinella* species and genotypes. Res Vet Sci.

[4] Pozio E (2000). Factors affecting the flow among domestic, synanthropic and sylvatic cycles of *Trichinella*. Vet Parasitol.

[5] Pozio E, Merialdi G, Licata E, Della Casa G, Fabiani M, Amati M (2020). Differences in larval survival and IgG response patterns in long-lasting infections by *Trichinella spiralis*, *Trichinella britovi* and *Trichinella pseudospiralis* in pigs. Parasit Vectors.

[6] Oksanen A, Interisano M, Isomursu M, Heikkinen P, Tonanzi D, Oivanen L (2018). *Trichinella spiralis* prevalence among wildlife of a boreal region rapidly reduced in the absence of spillover from the domestic cycle. Vet Parasitol.

[7] Murrell KD, Pozio E (2011). Worldwide occurrence and impact of human trichinellosis, 1986–2009. Emerg Infect Dis.

[8] Kapel CM (2000). Host diversity and biological characteristics of the *Trichinella* genotypes and their effect on transmission. Vet Parasitol.

[9] Kapel CM (2005). Changes in the EU legislation on *Trichinella* inspection-new challenges in the epidemiology. Vet Parasitol.

[10] Gajadhar AA, Forbes LB (2002). An internationally recognized quality assurance system for diagnostic parasitology in animal health and food safety, with example data on trichinellosis. Vet Parasitol.

[11] Gajadhar AA, Forbes LB (2010). A 10-year wildlife survey of 15 species of Canadian carnivores identifies new hosts or geographic locations for *Trichinella* genotypes T2, T4, T5, and T6. Vet Parasitol.

[12] Pozio E, Zarlenga D (2019). International commission on trichinellosis: recommendations for genotyping *Trichinella* muscle stage larvae. Food Waterborne Parasitol.

[13] Zarlenga DS, Chute MB, Martin A, Kapel CM (1999). A multiplex PCR for unequivocal differentiation of all encapsulated and non-encapsulated genotypes of *Trichinella*. Int J Parasitol.

[14] Reslova N, Skorpikova L, Slany M, Pozio E, Kasny M (2017). Fast and reliable differentiation of eight *Trichinella* species using a high resolution melting assay. Sci Rep.

[15] Sharma R, Harms NJ, Kukka PM, Jung TS, Parker SE, Ross S (2021). High prevalence, intensity, and genetic diversity of
* Trichinella
* spp. in wolverine (Gulo gulo) from Yukon, Canada. Parasit Vectors.

[16] Larter NC, Forbes LB, Elkin BT, Allaire DG (2011). Prevalence of
* Trichinella
* spp. in black bears, grizzly bears, and wolves in the Dehcho Region, Northwest Territories, Canada, including the first report of T.
* nativa
* in a grizzly bear from Canada. J Wildl Dis.

[17] Avramenko RW, Redman EM, Lewis R, Yazwinski TA, Wasmuth JD, Gilleard JS (2015). Exploring the gastrointestinal "nemabiome": deep amplicon sequencing to quantify the species composition of parasitic nematode communities. PLoS ONE.

[18] Avramenko RW, Bras A, Redman EM, Woodbury MR, Wagner B, Shury T (2018). High species diversity of trichostrongyle parasite communities within and between Western Canadian commercial and conservation bison herds revealed by nemabiome metabarcoding. Parasit Vectors.

[19] Poissant J, Gavriliuc S, Bellaw J, Redman EM, Avramenko RW, Robinson D (2021). A repeatable and quantitative DNA metabarcoding assay to characterize mixed strongyle infections in horses. Int J Parasitol.

[20] Watson SE, Hailer F, Lecomte N, Kafle P, Sharma R, Jenkins EJ (2020). Parasites of an Arctic scavenger; the wolverine (*Gulo gulo*). Int J Parasitol Parasites Wildl.

[21] Davey ML, Utaaker KS, Fossoy F (2021). Characterizing parasitic nematode faunas in faeces and soil using DNA metabarcoding. Parasit Vectors.

[22] Redman E, Queiroz C, Bartley DJ, Levy M, Avramenko RW, Gilleard JS (2019). Validation of ITS-2 rDNA nemabiome sequencing for ovine gastrointestinal nematodes and its application to a large scale survey of UK sheep farms. Vet Parasitol.

[23] Pafco B, Cizkova D, Kreisinger J, Hasegawa H, Vallo P, Shutt K (2018). Metabarcoding analysis of strongylid nematode diversity in two sympatric primate species. Sci Rep.

[24] Forbes LB, Gajadhar AA (1999). A validated *Trichinella* digestion assay and an associated sampling and quality assurance system for use in testing pork and horse meat. J Food Prot.

[25] Newman A (2014). Investigation of a human case of trichinellosis on a farm in southwest Ontario. Environ Health Rev.

[26] Scandrett B, Konecsni K, Lalonde L, Boireau P, Vallee I (2018). Detection of natural *Trichinella murrelli* and *Trichinella spiralis* infections in horses by routine post-slaughter food safety testing. Food Waterborne Parasitol.

[27] Landis JR, Koch GG (1977). The measurement of observer agreement for categorical data. Biometrics.

[28] Malakauskas A, Paulauskas V, Jarvis T, Keidans P, Eddi C, Kapel CM (2007). Molecular epidemiology of Trichinella spp. in three Baltic countries: Lithuania, Latvia, and Estonia. Parasitol Res.

[29] Franssen F, Deksne G, Esite Z, Havelaar A, Swart A, van der Giessen J (2014). Trend analysis of *Trichinella* in a red fox population from a low endemic area using a validated artificial digestion and sequential sieving technique. Vet Res.

[30] Dupouy-Camet J, Kociecka W, Bruschi F, Bolas-Fernandez F, Pozio E (2002). Opinion on the diagnosis and treatment of human trichinellosis. Expert Opin Pharmacother.

[31] Kumar V, Pozio E, de Borchgrave J, Mortelmans J, De Meurichy W (1990). Characterization of a *Trichinella* isolate from polar bear. Ann Soc Belg Med Trop.

[32] Kapel CM, Gamble HR (2000). Infectivity, persistence, and antibody response to domestic and sylvatic *Trichinella* spp. in experimentally infected pigs. Int J Parasitol.

[33] Castano C, Berlin A, Brandstrom Durling M, Ihrmark K, Lindahl BD, Stenlid J (2020). Optimized metabarcoding with Pacific biosciences enables semi-quantitative analysis of fungal communities. New Phytol.

[34] Lu KL, Nelson JO, Watase GJ, Warsinger-Pepe N, Yamashita YM (2018). Transgenerational dynamics of rDNA copy number in *Drosophila* male germline stem cells. Elife.

[35] Bik HM, Fournier D, Sung W, Bergeron RD, Thomas WK (2013). Intra-genomic variation in the ribosomal repeats of nematodes. PLoS ONE.

[36] Francioli D, Lentendu G, Lewin S, Kolb S (2021). DNA Metabarcoding for the characterization of terrestrial microbiota-pitfalls and solutions. Microorganisms.

[37] Erster O, Roth A, King R, Markovics A (2016). Molecular characterization of *Trichinella* species from wild animals in Israel. Vet Parasitol.

[38] Pozio E, Zarlenga DS (2013). New pieces of the *Trichinella* puzzle. Int J Parasitol.

[39] Caetano Wyler S, Naciri Y (2016). Evolutionary histories determine DNA barcoding success in vascular plants: seven case studies using intraspecific broad sampling of closely related species. BMC Evol Biol.

[40] D'Amore R, Ijaz UZ, Schirmer M, Kenny JG, Gregory R, Darby AC (2016). A comprehensive benchmarking study of protocols and sequencing platforms for 16S rRNA community profiling. BMC Genomics.

[41] Lerch A, Koepfli C, Hofmann NE, Messerli C, Wilcox S, Kattenberg JH (2017). Development of amplicon deep sequencing markers and data analysis pipeline for genotyping multi-clonal malaria infections. BMC Genomics.

