# Supplementary material for: Identification of Trichinella taxa by ITS-1 amplicon next-generation sequencing with an improved resolution for detecting underrepresented genotypes in mixed natural infections
Source: Parasit Vectors. 2023 Dec 21;16:466. doi: 10.1186/s13071-023-06035-1 (PMC10734138; doi:10.1186/s13071-023-06035-1)
Supplement: Supplementary file 1 — Additional file 1. Reference sequences of the ITS-1 marker fragment of Trichinella spp. [file 13071_2023_6035_MOESM1_ESM.docx]

>T. spiralis (T1)

CTGCGGAAGGATCATTATCGTATTTTTATACATGAAATGTGATTGATTTGTAATGTGTGC

AAAAAGAGCAGTTGCACTTTGTGTTGTGTGCAGTTGATCTTTGTTGTATTCATCCTCACT

ACTGATGCGTGAAAATGATGCTACATTCTTTCGCTCTGTGCAGAAAGGAAAATAATAAAT

CATTTGTAACGTGTATCATCAGTTGTGGTGGTTTTCTTTGTAATCCAGACACAATAAAAT

GACTTGATTTGTGTGTGTATATGCATGAATAAAGTCATCAGTGACGTTTGGTACCTGGGC

GTTCTGCGTCGATCTAATTTTAGCAGATCGATTGACGACTTGTGCAACATGACGGTT

>T. nativa (T2)

CTGCGGAAGGATCATTATCGTGTTTTCAAACATAAAAAGTGATTGTTTTGTGATGTGTGC

TAAAGAGCAGTTGCACTTTGTTGTGTGCAGTTGATCTTTGTATTCATCCTCACTGCTGAT

GCGTGAAAATGATGCTACATCCTTTTGATCTGTGCAAAAAGGAGATATAAATCATCTTTA

ACGCGGATTATCAGTTGTGATGGTTTTCTTTGTAATCCAGACACAATAAAATGACTTGAT

TTGTGTATTTGCATGAAGAAAAGTCATCAGTGACGTTTGGTACCTGGGCGTTCTGCGTCG

ATCTAATTTTTGCGGATCGATTGACGACTTGTGCAACATGACGGTT

>T. britovi (T3)

CTGCGGAAGGATCATTATCGTGTTTTCAAACGTAAAAAGTGATTGTTTTGTGATGTGTGC

TAAAGAGCAATTGCACTTTGTTATGTGCAGTGCTGATGTGTGTATTTATCCTCACTGCTG

ATGCGTGAAAATGATGCTACATCCTTTTGATCTGTTCAAAAAGGAGATTAATAAATCATT

TTTAACGCGGATTATCAGTTGTGATGGTTTTCTTTGTAATCCAGACACAATAAAATGACT

TGATTTGTGTATTTGCATGAAGAAAAGTCATCAGTGACGTTTGGTACCTGGGCGTTCTGC

GTCGATCTAATTTTTGCGGATCGATTGACGACTTGTGCAACATGACGGTT

>T. pseudospiralis (T4)

CTGCGGAAGGATCATTATCGTGTTTTCATTACATGAAAAGATCAAATTGTGTATTCTGCA

CAACAAAGAGGAGAAAAGCAGCAGCAATTGTGTTGTTCTCTTTTTTGTTCTGTGTAGAGT

TTTTTGGCCTCGCGACTGATGCGTGAAAATGCTGCCTCTTTTGCTGCTACACTAGTTAAT

AGTGGTAGTGCAAAAAAGGAGGGAATGAACAACGACAACTACATTAAAAAAGTAGTTGTT

GTTGTAGCATTGTAAACGCTGTCTGTCAGTTGTGTGGTGGTAATTGTACGTTTCAAACCA

GACACAATAAAATGACTCGATTTGTGTAAAAGCGCTTGCGCGTTGTTGCATAAAGACGTA

GTCATCAGTGACGTTTGGTACCTGCGCGTTCTGCGTCGATTTGTTCAAAAATTTTGGCGG

ATCGATTAACGACTTGTGCAACATGGCGGTT

>T. murrelli (T5)

CTGCGGAAGGATCATTATCGTGATTTCAAACGTAAAAAGTGATTGTTTTGTGATGTGTGC

TAAAGAGCAGTTGCACTTTGTTATGTGCAGTGCTGATGTGTGTATTTATCCTCACTGCTG

ATGCGTGAAAATGATGCTACATCCTTTTGATCTGTGCAAAAAGGAGATTAATAAATCATT

TTTAACGCGGATTATCAGTTGTGATGGTTTTCTTTGTAATCCAGACACAATAAAATGACT

TGATTTGTGTATTTGCATGAAGAAAAGTCATCAGTGACGTTTGGTACCTGGGCGTTCTGC

GTCGATCTAATTTTTGCGGATCGATTGACGACTTGTGCAACATGACGGTT

>Trichinella T6

CTGCGGAAGGATCATTATCGTGTATTCAAACATAAAAAGTGATTGTTTTGTGTGCTAAAA

AGCAGTTGCACTTTTTGTGCAGTGCTGATGTGTGTATTCATCCTCACTGCTGATGCGTGA

AAATGATGCTACATCCTTTTGATCTGTGCAAAAAGGAGATATAAATCATCTTTAACGCGG

ATTATCGGTAGTGATGGTTTTCTTTGTAATCCAGACACAATAAAATGACTTGATTTGTGT

ATTTGCATGAAGAAAAGTCATCAGTGACGTTTGGTACCTGGGCGTTCTGCGTCGATCTAA

TTTTTGCGGATCGATTAACGACTTGTGCAACATGACGGTT

>T. nelsoni (T7)

CTGCGGAAGGATCATTATCGTATTTTTATACATGAAAAGTAGTGATTCTTGTGTGCGCAA

TAAAGAGCAGTTGCACTTTGTTGTTGTATTCATCTTTACTGCTGCTACTGATGCGTGAAA

ATGATGTTACATCATTCATCCTTTTGCTCTGCTGTGCAAAAAGGGAAATAATAAGTCATT

TGTAACGCGTGGTATCAGTTGTGGTGGTTTTGTTTGTAATCCAGACACAATAAAATGACT

TGATTTTTGTGTATTTACATGAAGAAAAGTCATCAGTGACGTTTGGTACCTGGGCGTTCT

GCGTCGATCTGATTTTTTTGGATCGATTGACGACTTGTGCAACATGACGGTT

>Trichinella T8

CTGCGGAAGGATCATTATCGTGTTTTCAAACATAAAAAGTGATTGTTTTGTGATGTGTGC

ACTTTGTTGTGTGCAGTGCTGATGTGTATTTATCCTCACTGCTGATGCGTGAAAATGATG

CTACATCCTTTTGATCTGTGCAAAAAGGAGATTAATAAATCATTTGTAACGCGGATTATC

AGTTGTGATGGTTTTCTTTGTAATCCAGACACAATAAAATGACTTGATTTGTGTATTTGC

ATGAAGAAAAGTCATCAGTGACGTTTGGTACCTGGGCGTTCTGCGTCGATCTAATTTTTG

CGGATCGATTGACGACTTGTGCAACATGACGGTT

>Trichinella T9

CTGCGGAAGGATCATTATCGTGTTTTCAAACGTAAAAAGTGATTGTTTTGTGATGTGTGC

TAAAGAGCAGTTGCACTTTGTCATGTGCAGTGCTGATGTGTGTATTTATCCTCACTGCTG

ATGCGTGAAAATGATGCTACATCCTTTTGATCTGTGCAAAAAGGAGATTAATAAATCATT

TTTAACGCGGATTATCAGTTGTGATGGTTTTCTTTGTAATCCAGACACAATAAAATGACT

TGATTCGTGTATTTGCATGAAGAAAAGTCATCAGTGACGTTTGGTACCTGGGCGTTCTGC

GTCGATCTAATTTTTGCGGATCGATTGACGACTTGTGCAACATGACGGTT

>T. papuae (T10)

CTGCGGAAGGATCATTATCGTGTTACCAAACACAAAAAGTACTATACTTGAAAAGATGAT

ATTTTCTGCACAGGAGAGAAAGAGAATTTGACAAAGTTGTTCTTTTTCTTTGTGCGGGAG

TTGTTCTTTGCCTCATGACTGATGCGTGAAAATGCTGTTTCCTTTGCTGCAAGCGCAAAA

TGGAAAACATAACAACAACAACATGTTGTTGTGGCATTGTAAACGCTGACTATCAGTTGT

GGTGGTTGTTCGTGCAAAACCAGACACAATAAAATGACTCGATTTGTGTAAATACGTGTT

GTGTGTTTGCATGGAGAAAAGTCATCAGTGACGTTTGGTACCTGTGCGTTCTGCGTCGAG

CTGTTCAATTTTGGCGGCTCGACTAACGACTTGTGCAACATGACGGTT

>T. zimbabwensis (T11)

CTGCGGAAGGATCATTATCGTGTTACCGAACACAAAATACTATATACTTGAAAAGATGAT

ATTTTCTGCACAAGAGAGAAAGAGAATTTGACAATGTTGTTCTTTTTCTCTGTGCAGGAG

TGTTGTTCTTTGCCTCATGACTGATGCGTGAAAATGCTGTTTCTTTTGCTGCTGCTGCTG

CTGCTGCTGCTGCTGCAGCAAGCGCAAAATGGAAAAAATAACAACAACATCATGTTGTGG

CATTGTAAACGCTGACTATCAGTTGTGGTGGTTGATCGTGCAAAACCAGACACAATAAAA

TGACTCGATTTGTGTAAATACATGTGTGTTTGCATGGAGAAAAGTCATCAGTGACGTTTG

GTACCTGTGCGTTCTGCGTCGAACTGTTCAATTTTGGCGGCTCGACTAACGACTTGTGCA

ACATGACGGTT

>T. patagoniensis (T12)

CTGCGGAAGGATCATTATCGTATTTTTAAACATAAAACGTGATTGTTTTGTGGTGTGCTA

AAGAGCAGTTGCACTTTGTGTGCAGTGCTGATGTGTGTATTCATCCTCACTGCTGATGCG

TGAAAATGTAACATCCTTTTCATCTGTGCAAAAAGGAGAATAATAAATCATTTGTAACGC

GGATTATCAGCTGTGATGGTTTTCTTTGTAATCCAGACACAATAAAATGACTTGATTTGT

GTATTTGCATGAAGAAAAGTCATCAGTGACGTTTGGTACCTGGGCGTTCTGCGTCGATCT

AATTTTTGCGGATCGATTGACGACTTGTGCAACATGACGGTT

>T. chanchalensis (T13)

CTGCGGAAGGATCATTATCGTATTTTTAAACATAAAAAGTGATTTGTGATCGAAAGATCA

GTGCAATGCTGATGTGTGTATTCATCCTCACTGCTGATGCGTGAAAATGATGCTACGTCC

TTTTGATCTGTATAAAAGGAGATTTATAAATCATTTGTAACGCGGATTATCAGTCGTGAT

GGTTTTCTTTGTAATCCAGACACAATAAAATGACTTGATTTGTGTATTTGCATGAAGAAA

AGTCATCAGTGACGTTTGGTACCTGGGCGTTCTGCGTCGATCTAATTTTTGCGGGTCGAT

TGACGACTTGTGCAACATGACGGTT
